# Strumal carcinoid tumor of the ovary: A rare case report

**DOI:** 10.1097/MD.0000000000018009

**Published:** 2019-11-27

**Authors:** Wei Chai, Wenlei Zhang, Li Zhou, Xiaoyan Sun, Guifeng Jia

**Affiliations:** a Department of Gynecology and Obstetrics; b Department of Interventional Therapy; c Department of Radiology, The First Hospital of Jilin University, Changchun, Jilin, China.

**Keywords:** strumal carcinoid tumor of the ovary, ovary, carcinoid, thyroid

## Abstract

**Rationale:**

Strumal carcinoid tumor of the ovary (SCTO) is a very rare kind of ovarian tumor. The symptoms of SCTO are often nonspecific and misleading. Therefore, a full understanding of the characteristics, diagnosis, and treatment methods of SCTO is important.

**Patient concerns:**

In this study, we report a 63-year-old woman with SCTO whose most obvious symptoms were abdominal distention and weight loss of 5 kg for about 1 month.

**Diagnosis:**

Her carbohydrate antigen 125 (CA125) levels were higher than normal. Gynecologic sonography showed an 8.8 × 7.5 cm mass with mixed density and an irregular shape behind the uterus. Pathologic and immunohistologic examinations confirmed SCTO of the right ovary.

**Interventions:**

The patient underwent complete surgical resection of the whole uterus, bilateral ovaries, and fallopian tubes.

**Outcomes:**

The patient recovered well with no obvious complications and was discharged on the 10th day postsurgery.

**Lessons:**

Complete surgical resection is vital to treat SCTO. Postsurgical pathologic and immunohistologic examinations can confirm a diagnosis of SCTO.

## 1 Introduction

Carcinoids are a kind of neuroendocrine tumor that usually occurs in the gastrointestinal and respiratory systems. Primary ovarian carcinoid is a very rare ovarian tumor, accounting for 0.5% to 1.7% of all carcinoids and about 1% of ovarian cancer. Primary ovarian carcinoid is divided into 4 categories based on their histopathologic characteristics: insular, strumal, trabecular, and mucinous.^[1]^ Strumal carcinoid tumor of the ovary (SCTO) makes up 40% of primary ovarian carcinoid cases.^[2]^ SCTO is derived from the endoderm and always comprises thyroid tissue intimately mixed with the carcinoid tumor.^[1]^

In the present study, we report a rare case of SCTO and share our experience of the basic characteristics, diagnosis, and treatment methods of SCTO.

## 2 Case report

This study was approved by the Ethics Committee and institutional Review Board of our hospital. The patient provided informed consent for publication of the case.

**Figure 1:**
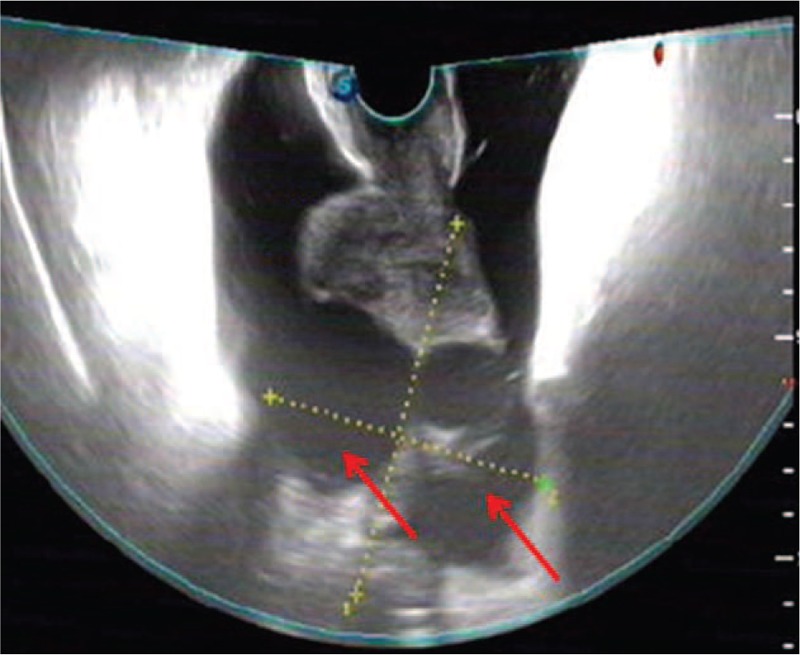
Gynecologic sonography showing an 8.8 × 7.5 cm mass (red arrow) with mixed density and irregular shape behind the uterus.

A 63-year-old woman attended the Department of Digestive Internal Medicine of our hospital with abdominal distention and weight loss of 5 kg for about 1 month. She denied any history of nausea, vomiting, cough and expectoration, or fecal abnormalities. In addition, the patient also denied a history of hypertension, coronary heart disease, diabetes, or acute and chronic infectious diseases. The physical examination showed that the patient's general condition was good and her vital life signs were stable; however, her abdomen was slightly distended with no tenderness, and no rebound tenderness or muscle tension. The shifting dullness of the patient was positive. Laboratory tests showed almost normal liver and thyroid function; however, the patient's level of carbohydrate antigen 125 (CA125) was 768.10 IU/mL, which was higher than the normal level (0–35 IU/mL). Abdominal computed tomography (CT) examination showed some liver calcification, multiple gallbladder stones, and a large amount of fluid accumulated in the abdominal and pelvic cavities. Furthermore, the CT scan also showed a 7.7 × 7.1 cm mass with a mixed density in the right side of the pelvis. Gynecologic sonography showed that the uterus was floating, was in the anterior place, and was about 3.9 cm × 3.1 cm in size with a normal shape. Gynecologic sonography also showed an 8.8 × 7.5 cm mass with a mixed density and irregular shape behind the uterus (Fig. 1). Based on these examination results, the clinicians in the Department of Digestive Internal Medicine considered that the mass in the pelvic cavity might be a malignant tumor of the ovary. Consequently, the patient was sent to the Department of Gynecology and Obstetrics for surgical treatment.

**Figure 2:**
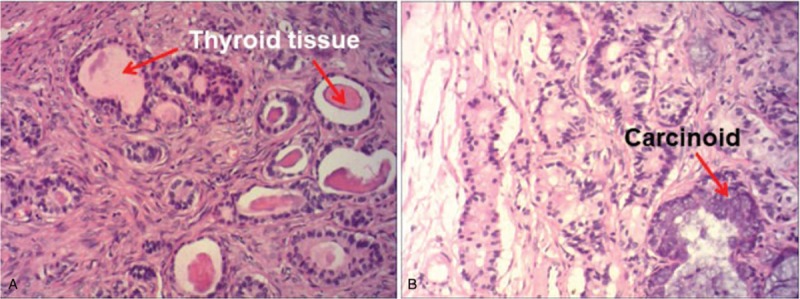
Histologic examination (hematoxylin and eosin staining) of the strumal carcinoid tumor of the ovary. Red arrows in (A) indicate the thyroid tissue and the red arrow in (B) indicates the carcinoid.

**Figure 3:**
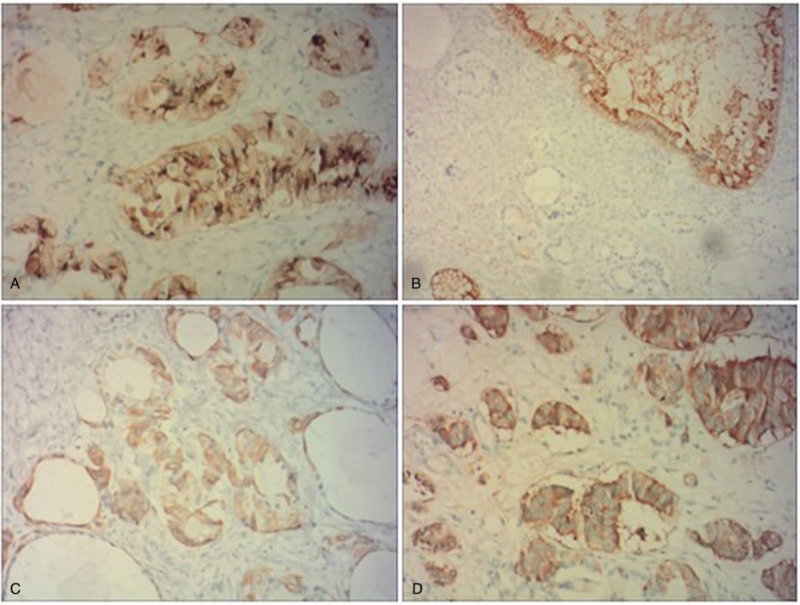
Immunohistologic staining of the strumal carcinoid tumor of the ovary. (A) CgA, (B) Galectin-3, (C) neuron-specific enolase, and (D) synaptophysin.

Exploratory laparotomy showed that the patient's right ovary was enlarged (8.0 cm × 7.0 cm in size) and the left ovary and the bilateral fallopian tubes were normal. The whole uterus, bilateral ovaries, and fallopian tubes were then resected and rapid pathologic examination indicated SCTO of the right ovary. The postsurgical pathologic examination showed thyroid adenomatous changes and carcinoid tumor changes in the right ovary (Fig. 2). Immunohistologic tests showed positive expression of chromogranin A (CgA), Galectin-3, neuron-specific enolase (NSE), and synaptophysin (Syn) (Fig. 3), which are biomarkers of carcinoid tumors. The patient recovered well and was discharged on the 10th day after surgery.

## 3 Discussion

Primary SCTO is very rare and accounts for <0.1% of ovarian cancer cases.^[3]^ There is no consensus concerning the tissue origin of SCTO. Researchers agree that SCTO comprises carcinoid and thyroid follicles and is a type of single layer teratoma.^[4]^ Most cases of SCTO occur in one side of the ovary; however, Zahradka and Schulz reported 1 case in which SCTO was found in both sides of the ovaries.^[5]^

Patients with SCTO always present with no obvious symptoms, and most of them attend the hospital because of lower abdominal masses that are found incidentally. Some larger SCTOs can result in different levels of abdominal pain. In our study, the patient denied an obvious history of abdominal pain; however, she had abdominal distention caused by a large volume of paracentesis.

Long-term constipation may be the main symptom in a small number of patients with SCTO. Motoyama et al identified large amounts of cells that could release peptide YY in SCTO tissues.^[6]^ Peptide YY can inhibit many intestinal functions, including the secretion of gastric acid, gastric emptying, the secretion of chloride from the small intestine and colon, and insulin secretion. Peptide YY also inhibits the movement of the jejunum and colon.^[4,7]^ As a result, the release of peptide YY is considered a possible explanation for the constipation symptoms of patients with SCTO. For female patients with constipation for unexplained reasons, SCTO should be considered.

Some patients with SCTO present with endocrine dysfunction. Robboy and Scully summarized 50 cases of SCTO, and found that 8% of the patients showed increased levels of steroid hormones, and the thyroid follicles of the tumor tissues played a role in thyroid function in these patients with SCTO.^[8]^ Ashton reported a case of SCTO with hyperinsulinemia, hypoglycemia, and pigmentation.^[9]^ The patient in our study showed no signs of endocrine dysfunction.

The SCTO has no typical clinical symptoms and no specific features in imageologic examinations. Therefore, a correct diagnosis of SCTO is difficult before surgery. High levels of CA125, which could lead to a large volume of paracentesis, are always found in patients with SCTO. However, higher CA125 levels are also a feature of malignant ovarian tumors.^[10]^ Pathologic and immunohistologic examinations are the gold standard for the diagnosis of SCTO. The pathologic examination always shows the thyroid adenomatous changes and signs of carcinoid tumor.^[11]^ The immunohistologic examination shows features of carcinoids, such as positive staining for CgA, Galectin-3, NSE, and Syn.^[12]^

Surgical treatment is the main therapeutic option for SCTO. For young women with SCTO, the affected ovary and fallopian tube should be resected. However, for patients with SCTO beyond reproductive age, the whole uterus, bilateral ovaries, and fallopian tubes could be resected.^[3,13,14]^ SCTO is often associated with teratomas, which can be malignant, and the thyroid components of SCTO can also become thyroid papillary or follicular carcinomas. In such cases, surgical treatment plus additional chemotherapy or radiotherapy should be applied to treat SCTO.

In the reported case, SCTO occurred in an elderly woman whose most obvious symptom was abdominal distention. Complete surgical resection of the whole uterus, bilateral ovaries, and fallopian tubes was performed. Pathologic and immunohistologic examinations confirmed a diagnosis of SCTO. Our study also discussed the basic characteristics, diagnosis, and treatment methods of SCTO.

## Author contributions

**Conceptualization:** Wei Chai.

**Data curation:** Wenlei Zhang.

**Formal analysis:** Li Zhou.

**Investigation:** Wenlei Zhang, Xiaoyan Sun.

**Project administration:** Li Zhou.

**Resources:** Wenlei Zhang, Guifeng Jia.

**Software:** Wenlei Zhang, Guifeng Jia.

**Validation:** Wei Chai, Guifeng Jia.

**Visualization:** Wei Chai.

**Writing – original draft:** Wei Chai.

**Writing – review & editing:** Xiaoyan Sun, Guifeng Jia.
